# Exploring efficient grouping algorithms in regular expression matching

**DOI:** 10.1371/journal.pone.0206068

**Published:** 2018-10-24

**Authors:** Chengcheng Xu, Jinshu Su, Shuhui Chen

**Affiliations:** 1 College of Computer, National University of Defense Technology, Changsha, Hunan, China; 2 National Key Laboratory for Parallel and Distributed Processing, Changsha, Hunan, China; Northeast Normal University, CHINA

## Abstract

**Background:**

Regular expression matching (REM) is widely employed as the major tool for deep packet inspection (DPI) applications. For automatic processing, the regular expression patterns need to be converted to a deterministic finite automata (DFA). However, with the ever-increasing scale and complexity of pattern sets, state explosion problem has brought a great challenge to the DFA based regular expression matching. Rule grouping is a direct method to solve the state explosion problem. The original rule set is divided into multiple disjoint groups, and each group is compiled to a separate DFA, thus to significantly restrain the severe state explosion problem when compiling all the rules to a single DFA.

**Objective:**

For practical implementation, the total number of DFA states should be as few as possible, thus the data structures of these DFAs can be deployed on fast on-chip memories for rapid access. In addition, to support fast pattern update in some applications, the time cost for grouping should be as small as possible. In this study, we aimed to propose an efficient grouping method, which generates as few states as possible with as little time overhead as possible.

**Methods:**

When compiling multiple patterns into a single DFA, the number of DFA states is usually greater than the total number of states when compiling each pattern to a separate DFA. This is mainly caused by the semantic overlaps among different rules. By quantifying the interaction values for each pair of rules, the rule grouping problem can be reduced to the maximum *k*-cut graph partitioning problem. Then, we propose a heuristic algorithm called the one-step greedy (OSG) algorithm to solve this NP-hard problem. What’s more, a subroutine named the heuristic initialization (HI) algorithm is devised to further optimize the grouping algorithms.

**Results:**

We employed three practical rule sets for the experimental evaluation. Results show that the OSG algorithm outperforms the state-of-the-art grouping solutions regarding both the total number of DFA states and time cost for grouping. The HI subroutine also demonstrates its significant optimization effect on the grouping algorithms.

**Conclusions:**

The DFA state explosion problem has became the most challenging issue in the regular expression matching applications. Rule grouping is a practical direction by dividing the original rule sets into multiple disjoint groups. In this paper, we investigate the current grouping solutions, and propose a compact and efficient grouping algorithm. Experiments conducted on practical rule sets demonstrate the superiority of our proposal.

## Introduction

Modern network services increasingly rely on the processing of stream payloads. These services leverage the features identified from the payloads to perform content-aware network applications, such as traffic billing, application protocol identification, load balancing, and network intrusion detection, etc. [1] Deep packet inspection (DPI) is the core component for the identification process, here the “deep” means the inspection checks not only the header part but also the payload part. In the DPI process, the packet payload is compared with hundreds of predefined signatures byte by byte, to detect out whether the payload matches any signature(s).

In the early days, signatures were mainly described with simple character strings. Classical string matching algorithms such as Aho-Corasick (AC) algorithm [2] and SBOM algorithm [3] could provide efficient matching speed with linear space consumption. With the development of network applications, exact strings were incompetent for signature description. Then, the regular expression is introduced as a main tool for its powerful and flexible description ability. Now the regular expression (RE) has been widely used in the network applications such as the Linux application protocol classifier (L7-filter) [4], the network intrusion detection system of Snort [5] and Bro [6], as well as the network devices such as the Cavium matching engines [7] and the IBM PowerEN processor [8].

For automatic processing on the platforms, the regular expression rules need to be converted to an equivalent finite state automaton (FSA) first. Each state in the FSA represent a different matching progress, and the matching process is an input byte driven state traversal process. The matching process starts from the initial state, in each step, the matching engine reads one byte from the payload sequentially. Based on the current state(s) and input byte, it inquires the FSA to achieve the next state(s). Then the achieved state(s) will be regarded as the current active state(s) for the processing of the next input byte. This process loops until the last byte of the payload. During the traversal process, any accessed final state denotes the identification of its corresponding regular expression rules.

There are two kinds of traditional FSAs, namely the nondeterministic finite automata (NFA) and the deterministic finite automata (DFA), and they are equivalent in the description ability. Usually the RE rules are first compiled to the NFA, then the NFA is converted to an equivalent DFA with the subset construction algorithm. The NFA and DFA have the exact opposite behavior on the space cost and matching efficiency. In the DFA matching procedure, there is only one active state at any time. Thus for each input symbol the matching engine only needs one memory access to achieve the next active state, and the time complexity is the fixed *O(1)* for each byte processing. While for the NFA matching procedure, a set of NFA states maybe active concurrently, thus the matching engine needs to inquire the NFA multiple times to achieve the next active NFA state set. In the worst case, all NFA states maybe active concurrently.

For the excellent matching efficiency of the DFA, it is much more widely used in the memory-centric architectures. However, the subset construction algorithm usually introduce much state expansion even state explosion for the DFA. In fact, each DFA state represents a set of NFA states which maybe active concurrently during matching. Thus, when converting a NFA with *n* states to a DFA, the number of DFA states could be 2^*n*^ in the worst case.

For practical implementation, the space cost of the DFA storage should be as small as possible. The time cost of processing a given payload is equal to the payload length times the memory access latency. As the payload length is fixed, for better performance the DFA should be deployed on fast on-chip memories, such as caches and SRAMs. However, the space of these memories is usually very small, thus reducing the space cost of DFA is the main issue for DFA based matching.

As countermeasures, current works mainly focus on compressing the space requirement of DFA. The main data structure of the DFA is a two-dimensional matrix, where the rows represent the DFA states and columns represent the input symbols. Each element in the matrix is called a transition which denotes the next state for the corresponding state and input symbol. Due to the deterministic features of DFA, there exists much redundancy among the transition. Compression operation can be implemented from different dimensions, such as the state merging solutions [9] on the state dimension and alphabet re-encoding proposals [10–13] on the input character dimension. These solutions perform well on simple and small RE sets, while most other research focuses on the transition compression aspect. D^2^FA [14] is the most representative one, and many transition compression algorithms [10, 15–18] are based on the D^2^FA. A representative D^2^FA based solution, such as the A-DFA [10] proposed by Becchi, can achieve a compression ratio of more than 90% with no more than two memory accesses on average for each byte processing.

With the ever-increasing complexity and scale of the RE rule set, the state explosion has became an inevitable problem, which usually makes the DFA unavailable on moderate platforms. Though these compression solutions are very efficient, they would be inapplicable in practice because most compression solutions rely on the original DFA, while on the other hand the DFA is unavailable.

In this work, we focus on the solutions to solve the state explosion problem. More precisely, we aim at the so-called rule grouping method which was firstly proposed by Yu [19]. In the rule grouping method, the RE rule sets are divided into *k* disjoint sets and compiled to *k* separate DFAs to avoid the state explosion. In this way, the *k* DFAs can work concurrently on the same stream, thus it is extremely suitable for parallel platforms such as multi-core processors, GPUs, etc. Furthermore, as the grouping result is a set of standard DFAs, all the above mentioned compression solutions can be combined with the grouping solutions to further reduce the space cost.

Unfortunately, Yu’s method [19] is quite inefficient as it usually costs much time especially when the rule set is large. However, somewhat surprisingly, since the invention of rule grouping, only few subsequent works have been found in the literature. It is worth noting that Rohrer also proposed a rule grouping method based on simulated annealing (SA) algorithm [20] but the resulting performance was not good enough compared with Yu’s method despite that it improved the grouping efficiency. Similar to the rule grouping idea, Luchaup [21] proposed a novel and efficient structure called DFA-trees to solve the state explosion problem. This work allows fitting large rulesets in small groups by repeatedly approximating grouped DFAs. To some extent, the DFA-trees method and rule grouping method are orthogonal. Our proposed grouping algorithms could be combined with Luchaup’s work to further reducing the grouping time and improving the grouping results during the DFA-trees construction.

### Our contributions

Based on some empirical assumptions [20], the rule grouping problem could be reduced to the maximum *k*-cut graph partitioning problem. As a corresponding solution, we then propose a compact and efficient algorithm called the one-step greedy (OSG) algorithm, which achieves desirable performance in both the space cost and time cost. Furthermore, we devise a subroutine called heuristic initialization (HI) algorithm. The HI algorithm can generate relatively good initial solutions, which could make significant improvements for these grouping algorithms. Experimental results demonstrate that our OSG algorithm outperforms other solutions in terms of the space cost and the time cost in most cases, and the HI algorithm also works well for the grouping algorithms.

## Analysis of the rule grouping problem

**Fig 1:**
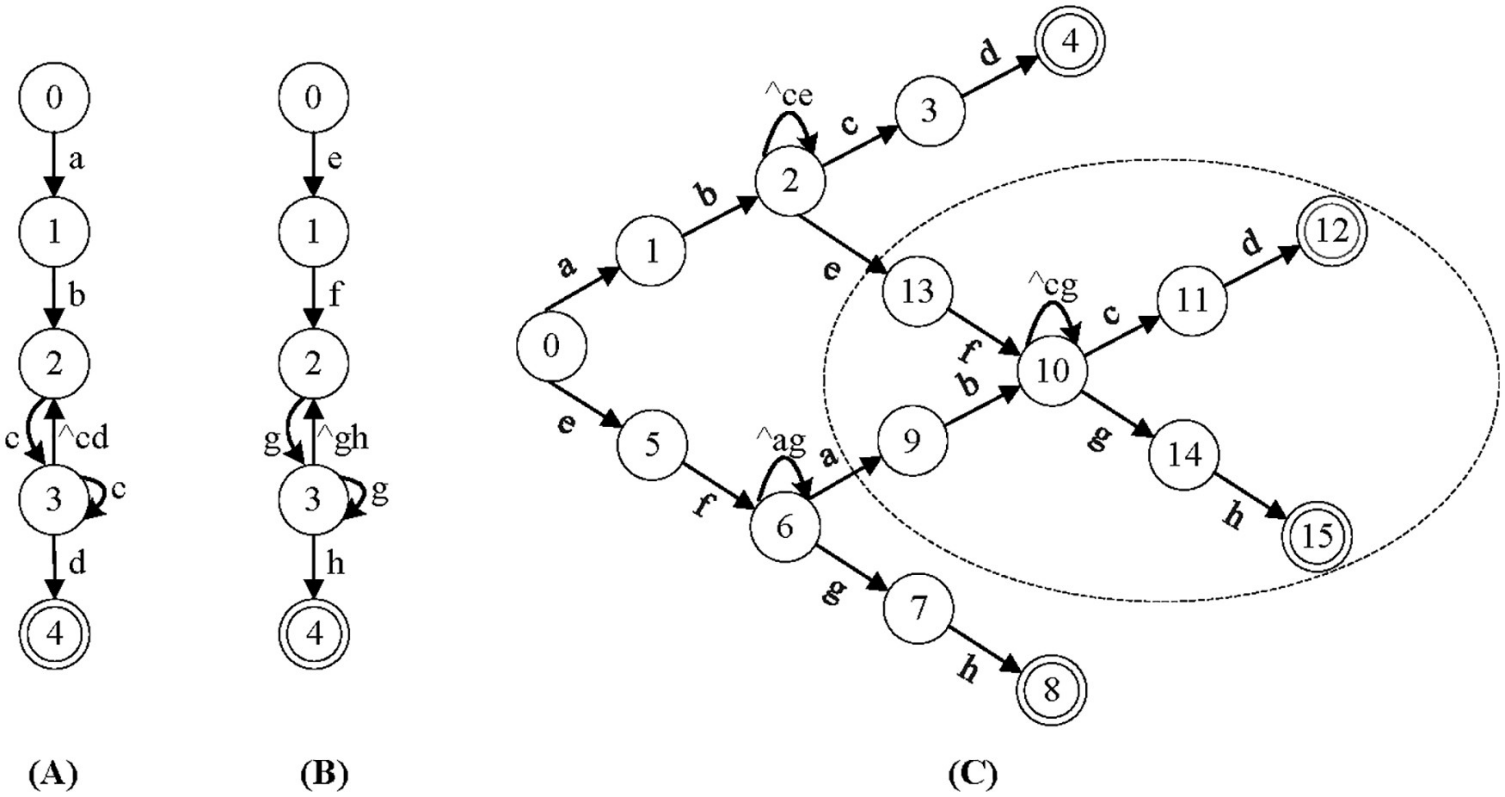
The illustration of the DFA state expansion caused by “.*”. (A) DFA for pattern “*ab*.*cd”, (B) DFA for pattern “*ef*.*gh”, (C) the composite DFA for the patterns of “*ab*.*cd” and “*ef*.*gh”. For simplicity, some transitions are omitted in these figures.

According to [22], DFA state explosion mainly raises from two kind of patterns, namely the patterns with “dot-star”-like features (such as “. *”, “. +”, “[^c]*”) and the patterns with “counting constraints” features (such as “.{n}”, “[^c]{n}”). A single RE with counting constraints may cause severe state expansion, but a single “dot-star” pattern never cause state expansion, the expansion arises only when compiling the “dot-star” pattern with other patterns together. Take the patterns “*ab*.*cd” and “*ef*.*gh” as example, there is no any expansion when compiling them separately, as shown in Fig 1A and 1B. But when compiling them together, state expansion occurs as shown in the ellipse of Fig 1C. As “.*” can match any sequence, extra states are needed to record the partial matching of these patterns. For example, state 10 represents both the prefixes “*ab*” and “*ef*” have been matched. When the number of “.*” rule grows, the combinations of the prefixes also increase exponentially, which would require huge number of DFA states.

Rule grouping is a natural method to restrain the expansion, because rules from different groups would not incur state expansion anymore. For a given RE set *RE*_*set*_ = {*r*_1_,*r*_2_, ⋯, *r*_*n*_}, rule grouping is to divide the rules into *k* disjoint subsets and compile each subset to a DFA. These DFAs can work concurrently on parallel platforms. For practical implementations, the time and space cost of grouping should be as small as possible.

Yu [19] defined the *interaction* as whether state expansion exists when compiling two rules together. Based on the interaction relationships of each pair of rules, Yu proposed a simple heuristic algorithm, and the main idea is to set an upper space limitation. As long as the current group does not exceed the limits, it chooses a rule which has least interactions with the current group, and adds the rule to the current group.

Yu only judges whether two rules have interaction. Rohrer [20] takes one step further, he also quantifies the interaction value. For the rule *r*_*i*_ and *r*_*j*_, *I*_*ij*_ in Eq (1) means the interaction value between *r*_*i*_ and *r*_*j*_, where *S* denotes the number of states when converting its subscripted rule(s) to a DFA. For example, *S*_*ij*_ means the corresponding number of states when compiling *r*_*i*_ and *r*_*j*_ to a DFA.

$$\begin{array}{c} I_{ij}=S_{ij}-S_{i}-S_{j} \end{array}$$ (1)

Empirical results reveal that, when adding a new rule *r*_*m*_ to the set *r*_*i*_ and *r*_*j*_, the increased number of DFA states can be estimated as *S*_*m*_ + *I*_*mi*_ + *I*_*mj*_, namely

$$\begin{array}{c} S_{ijm}=S_{ij}+S_{m}+I_{mi}+I_{mj} \end{array}$$ (2)

Combined with Eq (1), we can get Eq (3).

$$\begin{array}{c} S_{ijm}=S_{i}+S_{j}+S_{m}+I_{ij}+I_{mi}+I_{mj} \end{array}$$ (3)

Then for the *RE* set with *n* rules, we have

$$\begin{array}{c} S_{RE}=\sum_{i=1}^{n}S_{i}+\sum_{i=2}^{n}\sum_{j=1}^{i-1}I_{ij} \end{array}$$ (4)

When grouped into *k* subsets, the total number of states can be described as Eq (5), where *RE*_*l*_ denotes the *l*th group.

SRE=∑i=1nSi+∑l=1k∑i=2i∈REln∑j=1j∈REli-1Iij (5)

Now, our target is to find the best grouping solution to minimize the *S*_*RE*_. For a given rule set, the left item $\sum_{i=1}^{n}S_{i}$ is fixed, thus it is equivalent to minimize the right item of the plus sign. A direct method is to exploit every possible grouping solution, and choose the best one as the result. However, there exist *O*(*k*^*n*^) grouping solutions, the time cost is unacceptable even for several tens of rules.

Rohrer [20] further transforms the rule grouping to an equivalent graph partitioning problem. For a given RE rule set *RE*_*set*_, we can construct a weighted undirected graph *G* = (*V*, *E*), where each node represents a rule, and the edge weight denotes the interaction value for the corresponding pair of rules. Based on previous analysis, the problem is equivalent to find the graph partitioning solution whose total edge weight inside each group is minimum. As the total weight of the whole graph is fixed, it means that the total weight among groups for the solution should be maximum. This is a typical max k-cut graph partitioning problem, but it is NP-hard [23]. Therefore, it is very hard to find an optimal solution.

## Heuristic algorithms for the rule grouping problem

Wheeler [24] classified the solutions for max k-cut graph partitioning into three categories according to their effectiveness, namely the exact methods, the approximation methods with performance guarantees and the inexact methods without guarantees. The first two kinds of methods are too time-consuming to be applied in the rule grouping application, thus we employ the third kind of methods. Heuristic algorithms seem to be a possible direction for the rule grouping problem.

Through investigating research [25–27] for graph partitioning problem, we learned that the general combinational optimization algorithms such as simulated annealing (SA) algorithm and genetic algorithm (GA), etc. and the classical specific graph partitioning algorithm such as Kernighan-Lin (KL) method [28] are the most recommended algorithms. We have implemented these three algorithms, but we found that the grouping results of these algorithms are much worse than or even not comparable to the current best rule grouping algorithm [19]. Combined the above research and our evaluations, we learned that a given algorithm may have different degrees of adaptability to different applications or data sets. Thus, we are motivated to design new grouping methods for the specific rule grouping application. In this section, we devise a compact and efficient algorithm to handle the partitioning problem.

### Inspiration from the state expansion

Before explaining the one-step greedy algorithm, we would first introduce a subroutine called the heuristic initialization (HI) algorithm which is designed for improving the grouping algorithms. Note that the main purpose of rule grouping is to eliminate the interactions of rules from different groups. The “dot-star”-like characteristics such as “.*”, “.+”, “[^c]*” make a great contribution to state expansion, especially to the rule interactions. Different rules have different number of “.*”-like characteristics, thus they have different power to cause the state expansion. Intuitively, we should divide the rules with great expansion power into different groups as possible, in order to reduce the superposed expansion from different rules.

This provides us the inspiration to improve the grouping algorithms. For each rule *r*_*i*_, we use *EP*_*i*_ as in Eq (6) to quantify its expansion power. The *EP*_*i*_ can reflect the average expansion power of *r*_*i*_ when combining it with other rules.

$$\begin{array}{c} EP_{i}=\frac{1}{n}\sum_{j=1}^{n}\frac{I_{ij}}{S_{j}} \end{array}$$ (6)

With the assist of the quantified expansion power for each rule, we design a heuristic algorithm as in Algorithm 1 to generate an initial solution. This solution is not so good as the best grouping solution, but it is much better than a random grouping solution.

**Algorithm 1** The heuristic initialization algorithm

1: **for** *i* = 1 to *re*_*num* **do**

2:  Compute the expansion power *EP*_*i*_ for rule *r*_*i*_;

3: **end for**

4: Sort the *EP*s in descending order, assumed as $EP_{k_{1}},EP_{k_{2}},\cdots ,EP_{k_{re\_num}}$;

5: **for** *i* = 1 to *group_num* **do**

6:  Add rule ${\text{r}}_{k_{i}}$ to group *G_i_*;

7: **end for**

8: **for** *i* = *group*_*num* + 1 to *re*_*num* **do**

9:  Find the group with the least total *EP*, assumed as *G*_*j*_;

10:  Add rule ${\text{r}}_{k_{i}}$ to group *G*_*j*_;

11:  Update the total *EP* value for *G*_*j*_;

12: **end for**

13: return the current grouping solution;

The HI algorithm works as follows. First, the RE rules are sorted according to their expansion power in descending order (Step 1 to Step 4). Here, each subscript *k*_*i*_ denotes a specific rule identifier. Then, the first *k* rules are distributed to *k* groups separately (step 5 to step 7). For the remaining rules, in each step, the next rule is added to the group with the least expansion power, until all the rules have been grouped (step 8 to step 12). We will leverage the returned solution in step 13 to improve the grouping algorithms, with the expectation that a good initial solution would make contributions to the convergence rate and the final result.

**Fig 2:**
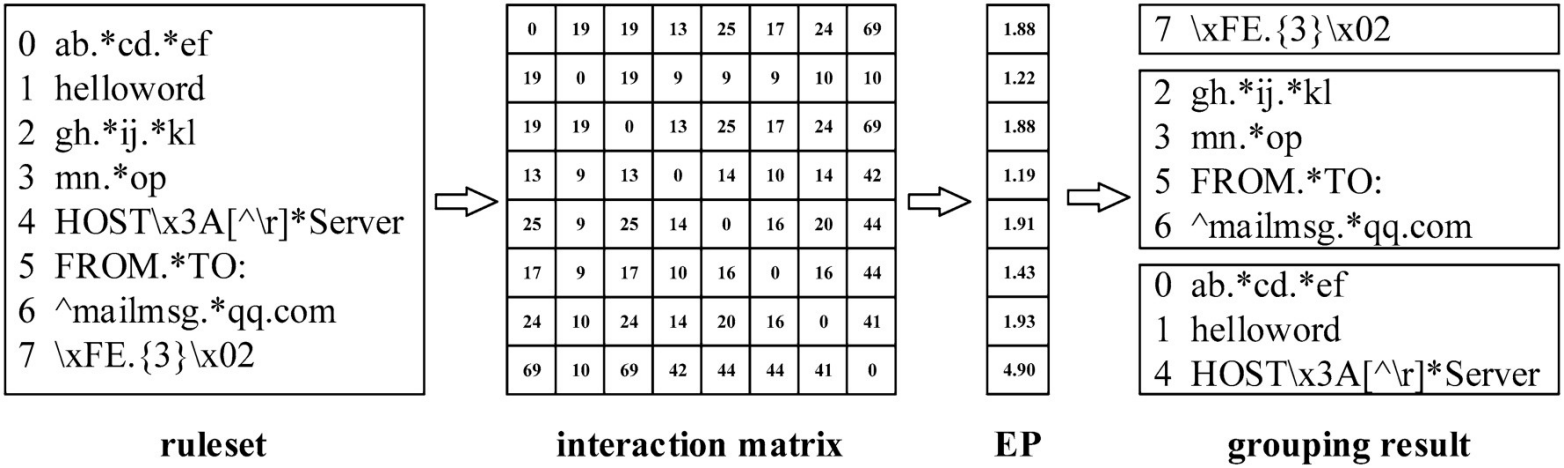
An example for the grouping procedures with the HI algorithm.

In order to better explain the HI algorithm, we present a simple example as shown in Fig 2. Suppose we need to divide 8 rules into 3 groups. With the given rules, first, we compute the interaction values for each pair of rules, as presented in the interaction matrix. Then, the average expansion power for each rule can be achieved as in the *EP* array with Eq (6). Finally, the *EP* array is ordered, and the rules are grouped according to Algorithm 1 (step 5 to step 12). The total state number of DFA would be 25503 if compiling these rules together, while after grouping the total state number of the grouped DFAs is only 443. This is a pretty good result, considering the simplicity and efficiency of the HI algorithm.

### The one-step greedy algorithm

The OSG in Algorithm 2 is a greedy algorithm for the rule grouping problem, and the core procedure is to search and execute the one-step move which provides the biggest gain, namely, the biggest cutsize increase. Here, the one-step move means moving a rule from its original group to another group. The OSG algorithm starts from a random initial solution. Then, it computes the cutsize increase matrix (step 2 to step 6), where each element *C*_*ij*_ denotes the increase of the total cutsize after moving rule *r*_*i*_ to group *G*_*j*_. This matrix is the main data structure in the grouping algorithm, and it will be updated along with the grouping process.

**Algorithm 2** The one-step greedy algorithm for the rule grouping problem

1: **Generate a random initial solution**;

2: **for** *i* = 1 to *re*_*num* **do**

3:  **for** *j* = 1 to *group*_*num* **do**

4:   Compute cutsize increase value *C*_*ij*_ if move rule *r*_*i*_ to group *G*_*j*_;

5:  **end for**

6: **end for**

7: **repeat**

8:  **repeat**

9:   Find the biggest cutsize increase value, assumed as *C*_*gh*_;

10:   Move the rule *r*_*g*_ to group *G*_*h*_;

11:   Mark the rule *r*_*g*_ as moved;

12:   Update all the cutsize increase values in *C*;

13:  **untill** every rule has been marked or all the cutsize are not positive

14:  Reset all rules as unmarked;

15: **untill** no more improvement

16: return the current solution;

The main process contains a nested loop. In the inner loop, each time it finds the one-step move with the biggest cutsize increase (step 9), and executes the corresponding move (step 10). Then the moved rule will be marked (step 11), and the matrix needs to be recomputed (step 12) due to the changes of the current grouping solution. The inner loop terminates until all rules have been moved or no positive cutsize increase exists in the matrix. While the outer loop works on the basis of the last inner loop, and it stops until no more improvements can be achieved with the execution of one-step move.

**Fig 3:**
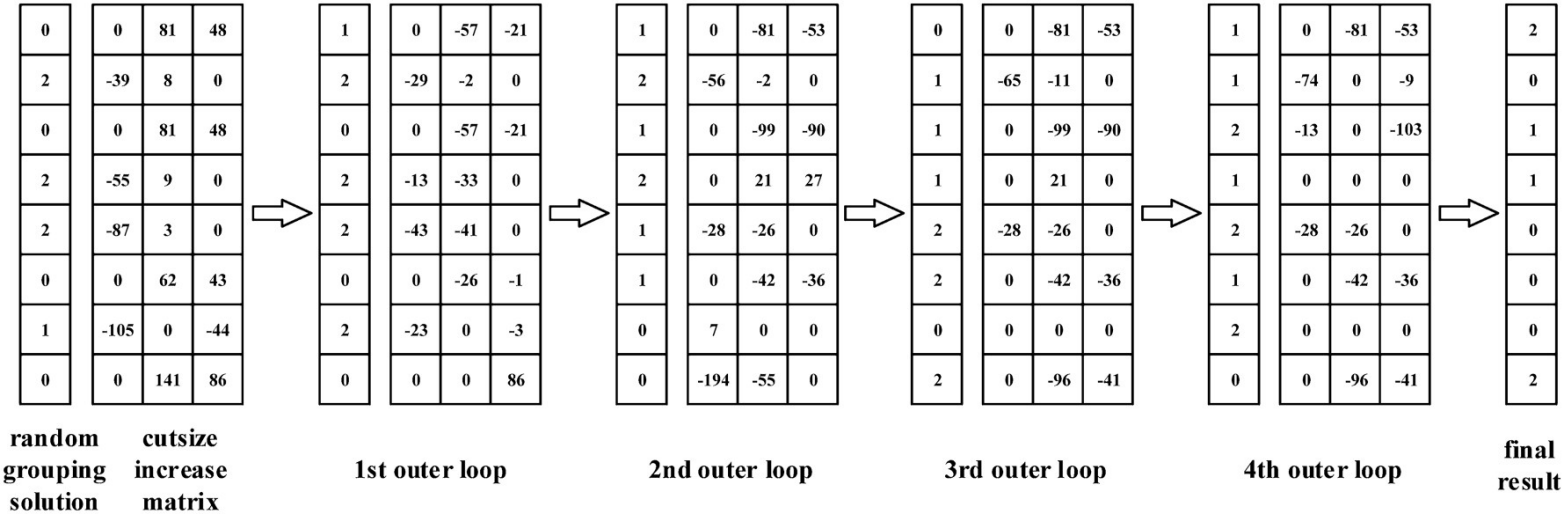
An example for the grouping procedures with the OSG algorithm.

Here, we also employ the ruleset in Fig 3 to illustrate how the OSG algorithm works for grouping. With an initial random grouping solution, it is easy to compute the cutsize increase matrix, where each element denotes the cutsize achievement when moving a corresponding rule to a corresponding group. The grouping process passes through 4 outer loops, and each outer loop involves several inner loop iterations, but for space limitation, we only present the results for outer loop iterations. In the inner loop, for each iteration, the algorithm searches for the one-step move with biggest cutsize achievement and executes this move. The inner loop ends until all the rules have been moved once or no positive one-step move can be found. Then for the next round of outer loop, all the rules will be set as unmoved. Finally, we get the grouping results as (1, 4, 5, 6; 2, 3; 0, 7), and the total state number of the grouped DFAs is 394.

Obviously, the one-step greedy strategy ensures that the result can always march forward to the better direction. We can simply prove this conclusion as follows.

*Proof*. The nested loop is the core of the OSG algorithm. For the outer loop, the initial solution of the current loop iteration is based on the returned solution of the previous loop iteration. The current loop iteration would not be adopted if it could not achieve a better solution than its previous loop iteration. Thus, each outer loop iteration yields a better at least not worse solution than the previous loop iteration. For the inner loop, the OSG algorithm repeats executing the one-step move which has biggest cutsize increase. Further, step 13 can guarantee that the value of the biggest cutsize increase in each loop iteration would be positive, which means that each loop iteration results in a bigger cutsize namely a better solution than the previous loop iteration. To sum up, the OSG algorithm would guarantee that the solution could always march forward to the better direction during loop iterations.

As all the actions of the OSG algorithm are based on the perspective of the simple one-step move, this may cause the omission of the global optimal solution. However, the outer loop of the OSG algorithm can guarantee that it would not fall into inferior local optima, because the outer loop will exploit any possible one-step move if this move can lead to a better solution.

## Experimental evaluation

Based on Becchi’s open-source RE compiler [29], we implemented the above mentioned algorithms. A random grouping algorithm is also implemented for comparison. Experiments are conducted on an Intel Xeon CPU E5-2630 (CPU: 2.3GHz, Memory: 32GB). Three RE rule sets were tested, including 109 RE rules from the L7-filter [4] protocol identification signatures, 127 RE rules from the backdoor file and 250 RE rules from the spyware-put file, the latter two sets are from the Snort [5] NIDS. As these rule sets are complex enough, none of them can be compiled to a single DFA on our platform.

For practical implementation in DPI applications, we divided each rule set into five to eight groups, and recorded the time cost for grouping and the space cost of the grouped DFAs for each algorithm. Except Yu’s algorithm, all other algorithms have random factors during the grouping procedure. Thus, a given algorithm would get various results for the same rule set. For reasonable comparisons, we repeated each of these algorithms ten times and compute the average time cost and space cost for comparison.

**Fig 4:**
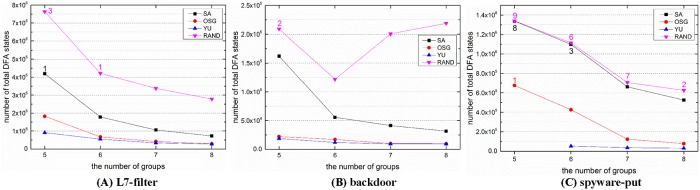
The space cost (total number of DFA states) for different solutions when dividing the rule sets into 5 to 8 groups. Each configuration was repeated ten times, and the figures reflect the average performance. The digital numbers beside the symbols in these figures denote the number of state explosions occurred during the ten trails.

**Fig 5:**
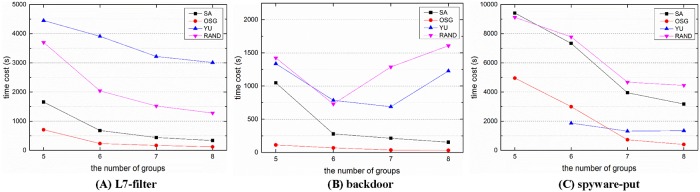
The time cost for different solutions when dividing the rule sets into 5 to 8 groups.

Figs 4 and 5 illustrate the space cost and time cost for different rule sets and algorithms. Even with grouping, the state explosion still happened, especially when the number of groups is small. We set 3 million states as the upper limit of a single DFA, the numbers beside the symbols in Fig 4 record the number of state explosions (the number of a single DFA states exceeds 3 million) occurred during the 10 runs. As the space cost and time cost are too huge, the explosions were terminated manually, and these situations were not counted for comparison.

For the space cost in Fig 4, it is obvious that Yu’s algorithm is superior to other algorithms in most situations, and our OSG algorithm is slightly worse than Yu’s algorithm but obviously better than the SA algorithm and the random algorithm. While on the other hand, the time cost of Yu’s algorithm is about 14 times higher than the OSG algorithm on average. It is because Yu’s algorithm requires lots of tests until it reaches the space limitation. And these tests would cost a lot of time for compilation. What’s more, the number of groups can not be set beforehand in Yu’s algorithm, thus the grouped number is unknown until the finish of grouping. For example, Yu’s algorithm could not divide the spyware-put rules into five groups after ten trials.

Yu’s algorithm is a classical grouping algorithm and achieves good grouping results. However, we still think that it is impractical to employ Yu’s algorithm in practice for the following reasons. First, the grouped DFAs are usually executed on parallel platforms. Each processing unit represents a matching engine and is responsible for matching a grouped DFA, and a packet is processed in parallel on these engines. Because the number of parallel units in a given platform is fixed, the number of grouped DFAs is preferably the same as (or a divisor of) the number of matching engines to maximize the use of the parallel platform. In Yu’s grouping algorithm, however, the number of grouped DFAs obtained by limiting the upper bound of the number of states in each group is uncertain and unpredictable. This will be very impractical because the engines are fixed in parallel platforms, and the uncertain number of groups would bring troubles for task scheduling. Second, the grouped DFA are deployed in the main memory, while the main memory is shared by all the matching engines. Our goal is that the overall space overhead is as small as possible and it is not usually required that the size of each grouped DFA should be similar. The same size for each grouped DFA does not make any sense for actual deployment and matching, so defining an upper limit on the number of states in each grouped DFA is not necessary.

Rohrer’s SA algorithm is the state of the art grouping method, but it does not perform as well as the OSG algorithm. SA’s space cost and time cost are separately 3.7 times and 4.4 times higher than the OSG algorithm on average. In addition, Rohrer’s SA algorithm encounters more state explosions in our experiments. The random grouping algorithm performs the worst, both in the space cost and time cost.

As a general optimization algorithm, the SA algorithm can be used to solve a broad range of problems. However, mapping a real problem to the domain of the SA algorithm could be difficult and it requires the familiarity with the algorithm [30]. To be specific, it involves the adjustment of a series of parameters and strategies, including how to determine an appropriate cooling strategy, how to perturb the current solution to generate the neighbor solution, how to select a proper random number generator, and how to implement the algorithm properly. The parameters depend on the specific data set. Thus to get better results, we need to constantly adjust the parameters for different data sets. In addition, both Johnson [31] and Williams [32] concluded that the SA algorithm can not perform very well for sparse graphs. In the domain of rule grouping, it means that the SA algorithm is not suitable for the rule set with many string-like patterns.

For the time cost in Fig 5, except Yu’s algorithm we can observe that the time cost has the same tendency with the corresponding space cost in Fig 4. This is because the time cost is mainly composed of the grouping procedure and the DFA compilation procedure after grouping, and the compilation plays a dominant role in the time cost.

**Fig 6:**
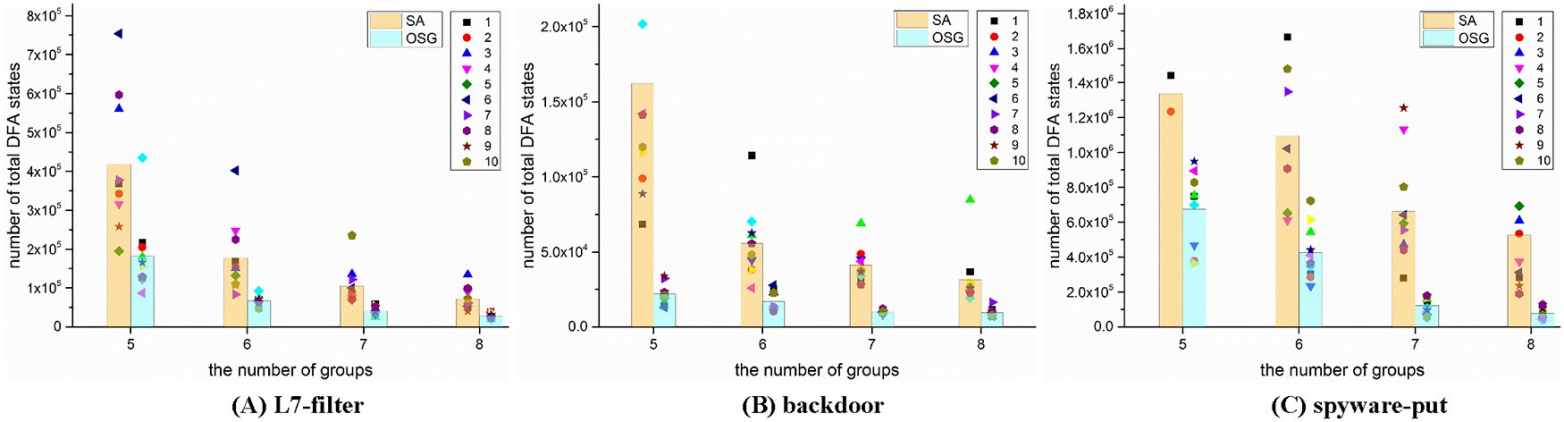
The comparison of grouping stability between the SA algorithm and the OSG algorithm. The column graphs reflect the average performance, and the symbols denote the specific grouping results.

As Rohrer’s SA algorithm is the state of the art method, we would make more comparisons between the SA algorithm and our OSG algorithm. Fig 6 displays the grouping results for ten different runs. We can observe that except for the better average space cost, the OSG algorithm also achieved better stability compared with the SA algorithm. In general, the space cost of the OSG algorithm is much more centralized than that of the SA algorithm. Even omitting the state explosion situations, the worst grouping result of the SA algorithm in spyware is even 6 times higher than its best one. We attribute the great variation in the SA algorithm to its various stochastic factors. There exist four random factors in the SA algorithm, namely the random initial grouping solution, the randomly selected neighbor numbers and positions, the random reset for neighbor solutions, and the uncertain probability to receive a bad neighbor solution. While the initial solution is the only random factor in the OSG algorithm. Thus the OSG algorithm has better stability on the whole.

**Fig 7:**
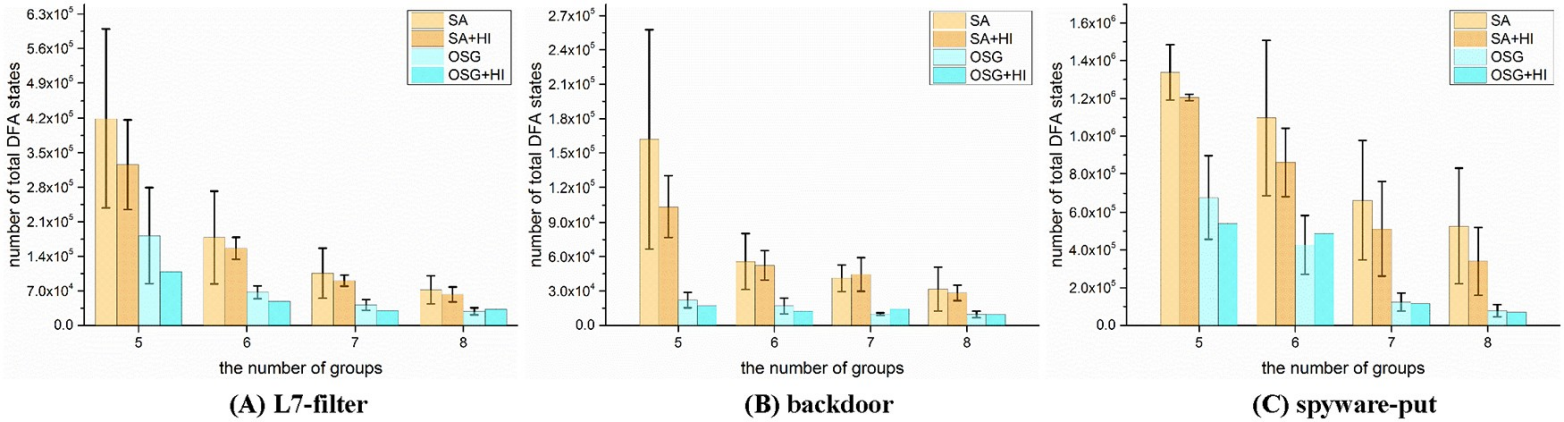
The HI’s effect on the space cost of the SA algorithm and the OSG algorithm. The vertical black lines in these figures represent the Y error lines for each configuration.

As the initial grouping solution is a main influencing factor for the grouping results, a better initial solution may yield better results. To verify this assumption, we employ the HI algorithm to generate good initial solutions, and use these solutions to optimize the grouping algorithms. We tested another ten runs for the SA and OSG algorithm with the initial solutions from HI, as shown in Fig 7. The vertical black lines are called Y error lines, which denote the range of standard deviation for each solution. It is obvious to find the great improvements, especially for the SA algorithm. The average space cost of SA separately dropped by 15%, 11% and 22% for the three rule sets. Another achievement is the improved stability, from the Y error lines, we can find that the grouping results are much more centralized after employing the HI subroutine. What’s more, with the HI’s support fewer state explosions occurred in the SA algorithm. As for the OSG algorithm, the space cost decreased generally except for few situations, and it separately saved 20%, 1% and 8% space for the three sets. In addition, as the only random factor is removed, the OSG algorithm is a deterministic algorithm now. On the other hand, as the HI subroutine did not involve the DFA compilation, the time cost would not increase much for the introduction of the HI subroutine.

## Conclusion

Rule grouping is a natural method to avoid the state explosion in regular expression matching. However, few research are found in this direction, and current solutions can not perform well in both the space cost and time cost. In this paper, we proposed a novel heuristic grouping algorithm called the OSG algorithm. Experiments show that our OSG algorithm outperforms the state-of-the-art algorithms. In addition, we also proposed a subroutine, called the HI algorithm, to improve the grouping algorithms with no more time overhead.

## Supporting information

S1 File The tested dataset of L7 file from L7-filter. (TXT)

S2 File The tested dataset of backdoor file from Snort NIDS. (TXT)

S3 File The tested dataset of spyware-put file from Snort NIDS. (TXT)

S4 File The achieved results and figures for these results in our experiment, this file should be opened with the Origin software. (OPJ)
